# Examining the Quasi-Static Uniaxial Compressive Behaviour of Commercial High-Performance Epoxy Matrices

**DOI:** 10.3390/polym15194022

**Published:** 2023-10-08

**Authors:** J. F. Gargiuli, G. Quino, R. Board, J. C. Griffith, M. S. P. Shaffer, R. S. Trask, I. Hamerton

**Affiliations:** 1 Bristol Composites Institute, School of Civil, Aerospace, and Design Engineering, Faculty of Science and Engineering, University of Bristol, Queen’s Building, University Walk, Bristol BS8 1TR, UK; joseph.gargiuli@bristol.ac.uk (J.F.G.); g.quino-quispe@imperial.ac.uk (G.Q.); rachel.board@bristol.ac.uk (R.B.); james.griffith@bristol.ac.uk (J.C.G.); r.s.trask@bristol.ac.uk (R.S.T.); 2 Department of Aeronautics, Imperial College London, South Kensington Campus, London SW7 2AZ, UK; 3 Department of Materials and Department of Chemistry, Imperial College London, South Kensington Campus, London SW7 2AZ, UK; m.shaffer@imperial.ac.uk

**Keywords:** composites, epoxy resins, compression, dilation angle

## Abstract

Four commercial high-performance aerospace aromatic epoxy matrices, CYCOM^®^890, CYCOM^®^977-2, PR520, and PRISM EP2400, were cured to a standardised 2 h, 180 °C cure cycle and evaluated in quasi-static uniaxial compression, as well as by dynamic scanning calorimetry (DSC) and thermogravimetric analysis (TGA). The thermoplastic toughened CYCOM^®^977-2 formulation displayed an overall increase in true axial stress values across the entire stress–strain curve relative to the baseline CYCOM^®^890 sample. The particle-toughened PR520 sample exhibited an overall decrease in true axial stress values past the yield point of the material. The PRISM EP2400 resin, with combined toughening agents, led to true axial stress values across the entire plastic region of the stress–strain curve, which were in line with the stress values observed with the CYCOM^®^890 material. Interestingly, for all formulations, the dilation angles (associated with the volume change during plastic deformation), recorded at 0.3 plastic strain, were close to 0°, with the variations reflecting the polymer structure. Compression data collected for this series of commercial epoxy resins are in broad agreement with a selection of model epoxy resins based on di- and tetra-functional monomers, cured with polyamines or dicarboxylic anhydrides. However, the fully formulated resins demonstrate a significantly higher compressive modulus than the model resins, albeit at the expense of yield stress.

## 1. Introduction

Advanced composites materials are used in a wide range of high-performance applications, from wind energy to automotive, as well as in the commercial and military aerospace sectors. The tensile properties of unidirectional fibre-reinforced polymer (FRP) composite materials are driven by the mechanical properties of the reinforcing fibres, while the compression performance is more significantly influenced by the mechanical properties of the polymer matrix [1,2]. Consequently, to develop more resilient composite materials in compression, it is important to characterise the compressive behaviour of commercial polymer matrices. The vast majority of high-performance matrices used in modern composite materials are based on patent-protected, proprietary epoxy resin formulations. In order to reach the desired high mechanical performance, tension/compression or fracture resistance (typically measured as the critical stress intensity factor, *K_IC_*), these epoxy matrices tend to include a range of toughening agents that can be organic or inorganic in their composition. Typical examples include both miscible thermoplastic adducts and discrete particulate fillers dispersed within the epoxy matrices. More specifically, epoxy formulations may be toughened using strategies ranging from amine- or epoxy- functionalised materials, such as carboxyl-terminated butadiene nitrile (CTBN) rubber adducts or phenoxy particles, or non-functionalised phase-separating thermoplastic polymers, such as polyethersulfone (PES), to the use of dispersed, micronised toughening particles, such as core-shell rubbers (CSR) as well as micro- or nano-fillers, such as silica, alumina, titanium dioxide, silicon carbide, carbon nanotubes, or graphene, with a variety of particle sizes and geometries [3,4].

Previous studies have focussed on the impact of the addition of toughening agents on the fracture toughness of model epoxy resins [5,6,7,8], as well as on the dynamic and quasi-static uniaxial compression performance of toughened and untoughened epoxy matrices [9,10,11,12,13,14]. These studies are mostly based on the modification of low-viscosity, untoughened, commercial difunctional epoxy resins derived from the diglycidyl ethers of bisphenol A (DGEBA) or bisphenol F (DGEBF). However, there is a dearth of available literature focussing on the quasi-static uniaxial compressive performance of formulated, toughened, commercial epoxy matrices. Realistic values are essential for understanding the performance of practical composites and calibrating computational models.

Furthermore, to date, little effort has been devoted to exploring the potential links between compression properties and dilation angle [15,16,17], although the volumetric change during plastic deformation of the matrix is likely to be important. These questions form the basis of the current systematic study, carried out on a series of chemically related epoxy resins, sourced from a unique supplier. The performance of these formulations was investigated in quasi-static uniaxial compression.

## 2. Materials and Methods

### 2.1. Cured Epoxy Materials

Four aromatic amine-aromatic epoxy formulations were cured to a standardised 2 h, 180 °C cure cycle and provided by Solvay (Wrexham, UK) as solid, cured cylindrical rods, typically 14 mm in diameter and 120 mm in height. CYCOM^®^890 (A) is a one-part aerospace epoxy resin designed for resin transfer moulding formulation and provides a baseline system for the study. CYCOM^®^977-2 (B) is a one-part aerospace epoxy resin formulation incorporating a novel epoxy compatible thermoplastic as its sole toughening agent. PR520 (C) is a one-part particulate-toughened epoxy resin formulation. PRISM EP2400 (D) is a single component epoxy resin formulation with a combination of toughening agents.

### 2.2. Simultaneous Thermal Analysis (STA)

Dynamic scanning calorimetry (DSC) and thermogravimetric analysis (TGA) were performed simultaneously using an STA (simultaneous thermal analysis) 449 F3 Jupiter, purchased from Netzsch (Selb, Germany). The simultaneous thermal analyses were performed on cured resin samples (12 mg ± 2 mg) in alumina crucibles from 50 to 800 °C, using a temperature ramp rate of 10 K/min., under a constant stream of nitrogen gas flowing through the furnace at a rate of 50 mL/min. Glass transition temperatures (*T_g_*, midpoint values) of the cured epoxy resins were determined using the DSC data recorded at temperatures below 250 °C. Degradation temperatures and mass losses of the various cured epoxy matrices were determined using the thermogravimetric data that were recorded between 50 and 800 °C.

### 2.3. Preparation of Compression Test Specimens 

**Figure 1:**
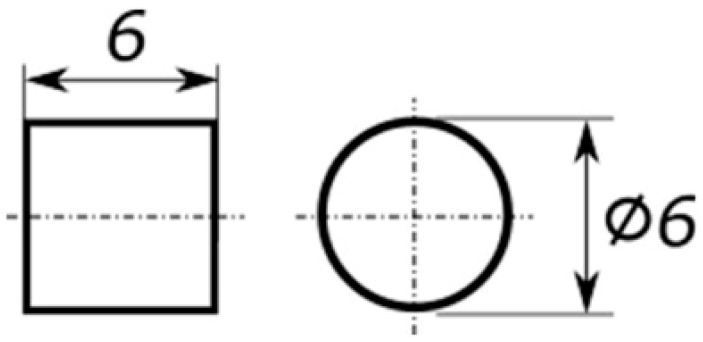
Dimensions of the compression specimens (mm).

The supplied cured epoxy rods were machined into plain cylindrical compression specimens (six specimens being tested per epoxy resin formulation to achieve statistically representative results), with a 1:1 (length/diameter, L/D) aspect ratio, as described in a previous study [18], using a diameter of 6.0 ± 0.1 mm and a height of 6.0 ± 0.1 mm (Figure 1). This 1:1 aspect ratio geometry was found to display less barrelling in comparison to longer aspect ratios [19,20], improving the stress uniaxiality of the experiments at large deformations. 

The test procedure was derived from the ASTM D695 international standard [21]. Specimens were dried in an air-vented oven at 50 °C for 2 days, according to the procedure described in ASTM D618 [22]. After conditioning, the specimens were stored inside a sealed desiccator, prior to being primed and speckled. The dry-machined cylindrical compression specimens were spray-painted individually using an acrylic primer matt white spray paint. Once the paint had dried, randomly scattered, black speckles (with an approximate speckle size of 100 μm) were applied over the entire surface of each of the specimens. This pattern was achieved by spraying, with an air brush, some India ink—a commercially available water-based colloidal carbon black ink. The speckled test specimens were then dried for about 1 h, at room temperature, in air, before being tested.

### 2.4. Quasi-Static Uniaxial Compression Testing

Uniaxial quasi-static compression tests were performed at room temperature, between 23 °C and 25 °C, with a constant vertical displacement rate of 0.36 mm/min., which corresponded to an initial strain rate in compression of 0.001 s^−1^. All compression tests were performed on a Shimadzu universal mechanical test frame, AGS-10 kN XD (Milton Keynes, UK), fitted with a 10 kN load cell and a pair of stainless steel cylindrical compression plates, each with a diameter of 30 mm. Each specimen was tested up to the limit of the calibrated load cell, i.e. 9.9 kN. The top and bottom plates were covered with a PTFE adhesive film (3M PTFE film tape 5490, with a film thickness of 90 μm) in order to reduce friction between the upper and lower faces of the cylindrical test specimens and the flat stainless steel compression plates they were in direct contact with, thus virtually eliminating any undesirable friction-induced deformations, such as barrelling, of the test specimens, at high axial true-strain values. The two PTFE films were replaced at the end of each test, before loading a new test specimen. At least 5 specimens of each material were tested.

### 2.5. Digital Image Correlation

**Figure 2:**
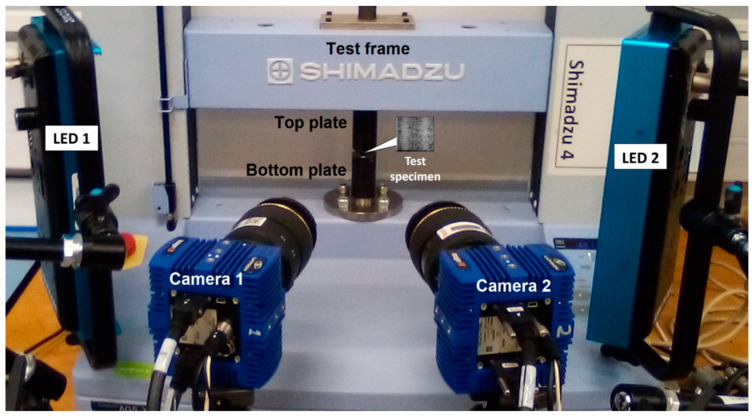
Compression and digital image correlation (DIC) experimental set-up showing position of compression test specimen (shown in expanded image).

Greyscale images of the speckled specimens were captured, at a frequency of 1 Hz, by a pair of DaVis LaVision 16MP CMOS digital image correlation (DIC) cameras (Bicester, UK), positioned symmetrically at an angle of 12° to the test specimen, as shown in Figure 2. After calibration and acquisition, images were analysed via the DaVis10 LaVision DIC software and synchronised with the load (F, in Newtons) analogue ± 5 V output of the Shimadzu universal test frame. The compression test specimens were illuminated by a pair of LED light arrays throughout the entire duration of the tests.

### 2.6. Data Reduction

The data reduction process is described below. Strains and stresses reported and discussed in this manuscript are true (i.e., logarithmic) strains and true stresses. The engineering strains $\varepsilon_{e}$ were obtained via DIC analysis and a representative virtual strain gauge (VSG) (36 pixels × 36 pixels), placed at the centre of the generated strain map, provided the values for instantaneous engineering radial and axial strain values, these values being correlated to instantaneous load values, acquired from the Shimadzu 10 kN load cell. For each set of correlated recorded images, true axial strain values (ε) were calculated using Equation (1).

(1) $$\varepsilon =\ln(\varepsilon_{e}+1)$$

True axial stress (*σ*) values were calculated by dividing the measured load *F* by the deformed cross-sectional area (also obtained from DIC).

The values for true axial stress were plotted against the true axial strain values. The resulting stress–strain curves were used to determine the compression modulus (*E*), Poisson’s ratio, and yield point. The yield point was defined in ASTM D695 as the point on the curve where stress values stop increasing with increasing strain values (local maximum). At that point, yield stress, *σ_y_*, and yield strain, *ε_y_*, were determined graphically for each of the four tested epoxy matrices. The elastic modulus was measured from the initial linear part of the axial strain vs. stress curves, via a linear regression within the axial strain range, within the linear response. The Poisson’s ratio (*ν*) was determined as the value of the slope of *ε*_xx_ against *ε_yy_*, before the yield point was reached; i.e., within the elastic region of the stress–strain plots.

Dilation angles (*ψ*), a measure of the amount of the volumetric strain within a tested material undergoing plastic deformation, were calculated using Equation (2), where *ν_p_* is the plastic Poisson’s ratio; i.e., the value of the Poisson’s ratio in the plastic region of the stress–strain curve.

(2) $$\psi =\tan^{-1}(-\frac{3(1-2\nu_{p})}{2(1+\nu_{p})})$$

For each tested material, dilation angle (*ψ*) values were plotted against true plastic axial strain values.

## 3. Results and Discussion

### 3.1. Simultaneous Thermal Analysis (STA)

**Figure 3:**
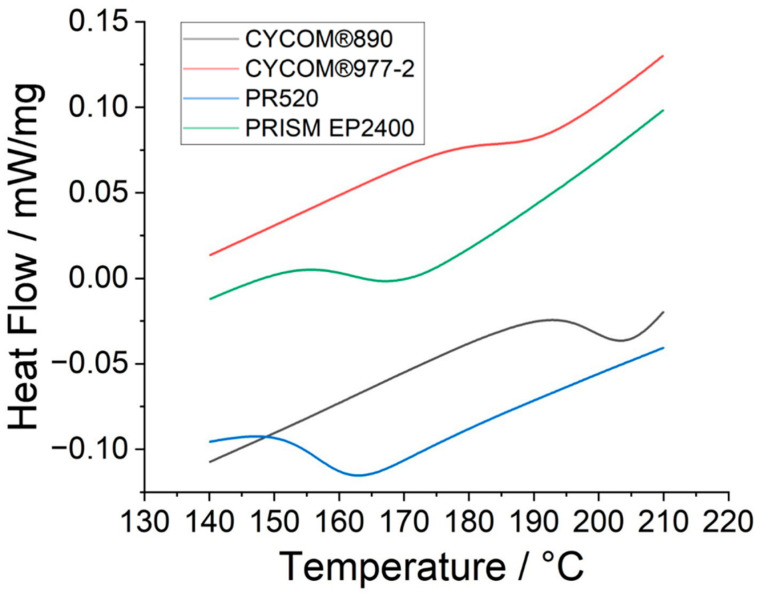
Differential scanning calorimetry of epoxy resins CYCOM^®^890 (A), CYCOM^®^977-2 (B), PR520 (C), and PRISM EP2400 (D) (exothermic processes shown upwards).

**Figure 4:**
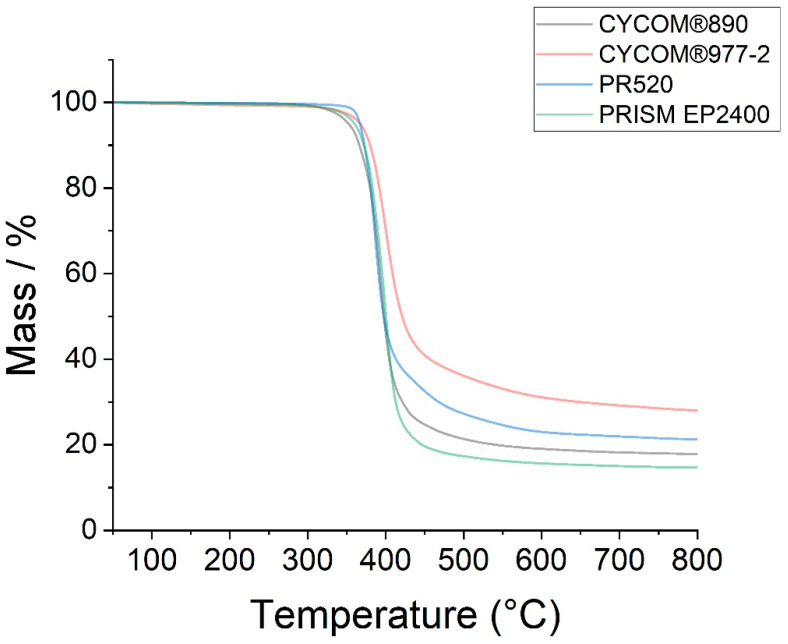
Thermogravimetric analysis of the degradation temperatures of epoxy resins CYCOM^®^890 (A), CYCOM^®^977-2 (B), PR520 (C), and PRISM EP2400 (D).

The DSC data (Figure 3) and TGA data (Figure 4) for the various epoxy resins are summarised in Table 1. Note, representative examples for the DSC (Figure S1) and TGA (Figure S2) are supplied in Supplementary Information to demonstrate how the respective *T_g_* (mid-point values), decomposition temperature (*T_dec_*), and total mass loss for the various epoxy resins were obtained from the raw data.

The TGA data show similarities (within the 3% tolerance on STA measurements) in the mass loss vs. the temperature profile for PRISM EP2400 (D) and CYCOM^®^977-2 (B), in their respective decomposition temperatures compared to CYCOM^®^890 (A) and PR520 (C). Similarly, there was no significant change in the recorded mass losses (within the 3% tolerance) between the baseline matrix CYCOM^®^890 (A) and the PR520 (C) or PRISM EP2400 (D). However, CYCOM^®^977-2 (B) showed a greater mass loss compared to the other three tested matrices.

The average critical stress intensity factor, *K_IC_* values, a measure of the susceptibility to cracking or the brittleness of the material (i.e., the crack propagation resistance) of the neat resins have been measured according to ASTM D5045 [23] and previously reported [24] by the manufacturer so that the baseline resin CYCOM^®^890 (A) yields a *K_IC_* value of 0.9 MPa m^0.5^, CYCOM^®^977-2 (B) has a value of 1.34 MPa m^0.5^, PR520 (C) has a value of 1.80 MPa m^0.5^, and PRISM EP2400 (D) produces a *K_IC_* value of 0.96 MPa m^0.5^. The *T_g_* values measured for the cured resins (in Table 1) are primarily affected by the chemical structures (and thus the network architecture) of the base resins and reflect the similarities between CYCOM^®^890 (A) and CYCOM^®^977-2 (B), which are chemically dissimilar from PR520 (C) and PRISM EP2400 (D). These data are consistent with the measured values for the manufacturer’s commercial datasheets for the resin systems [24] and with other systems previously reported in the literature [25].

### 3.2. Quasi-Static Uniaxial Compression Tests

**Figure 5:**
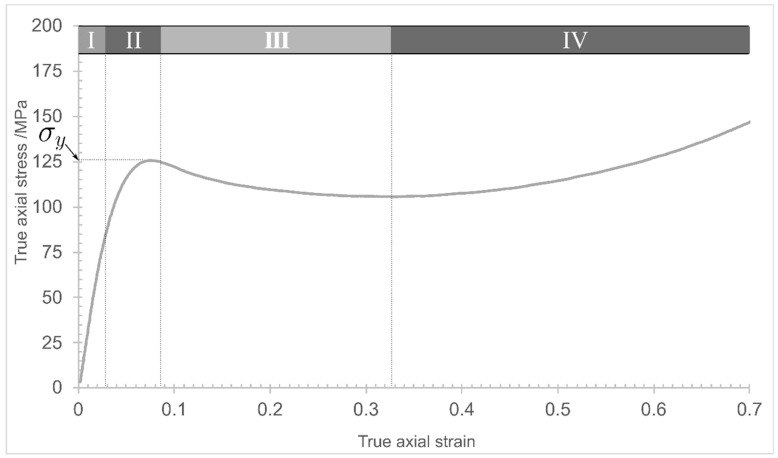
Representative stress–strain compression curve for an epoxy resin (redrawn from [10]).

The values for true axial stress were plotted against the true axial strain values. The resulting stress–strain curves were used to determine the compression modulus (*E*) as the slope from the linear portion of the elastic region of the curve. At that point, the yield stress, *σ_y_*, was determined and reported for each of the four tested epoxy matrices. This behaviour has been depicted by Morelle et al. [10], indicating that the true stress–true strain response for all materials follows five distinct stages, as depicted in Figure 5: (I) an initial linear stage corresponding to the material’s viscoelastic behaviour; (II) a nonlinear stage corresponding to the yielding of the material, which reaches a maximum value at the peak yield point; (III) a strain-softening stage following the yielding, and (IV) further strain hardening. The fifth stage, not depicted here, is either fracture for the quasi-static strain rates or unloading for the high strain rates. 

**Figure 6:**
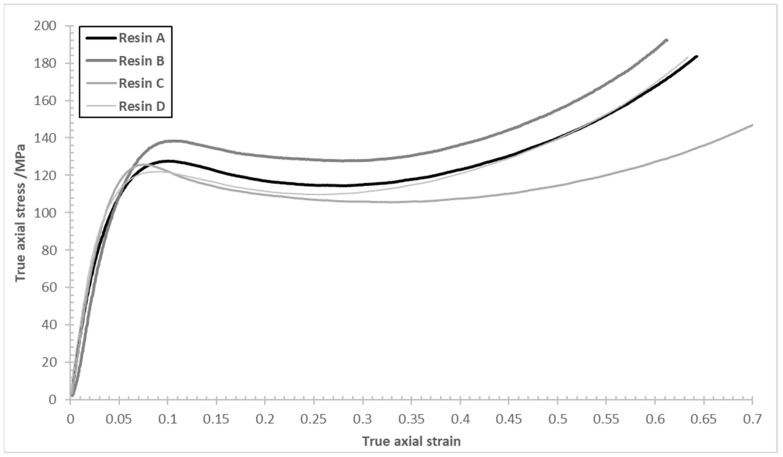
Stress–strain compression plots for epoxy resins CYCOM^®^890 (A), CYCOM^®^ 977-2 (B), PR520 (C), and PRISM EP2400 (D).

True axial stress vs. true axial strain curves for the four tested epoxy resins were determined in compression (Figure 6). The respective measured values of the compression modulus, *E*, yield stress, *σ_y_*, and yield strain, *ε_y_*, for the four tested epoxy matrices, are summarised in Table 2.

It is possible to compare the compression data gathered here with those of Elmahdy et al. [11], who studied the effects of nano-fillers on the compressive behaviour of HexFlow^®^RTM6 (similarly a one-component, untoughened epoxy resin designed for infusion processing). The latter is a comparatively well-known epoxy resin (comprising tetraglycidyl methylene dianiline, EEW = 116 g/eq, and two hardeners: 4,4-methylene*bis*(2,6-diethylaniline) and 4,4-methylenebis(2-isopropyl-6-methylaniline)). When cured to a high conversion, the high crosslink density makes RTM6 a comparatively brittle resin and, at the same initial strain rate in compression (0.001 s^−1^), the neat resin yielded an average compressive modulus of 3096 ± 47 MPa and a yield strength of 136 ± 6.21 MPa, with a Poisson’s ratio of 0.32 ± 0.126. These values are consistent, within error, to the cured epoxies in the present study.

The focus of the study by Elmahdy et al. was to investigate the effect of strain rate and filler content on the compressive behaviour of RTM6-based nanocomposites through the addition of silica nanoparticles with different sizes, weight concentrations, and surface functionalization. For the same strain rate, the best properties were obtained for functionalised nanosilica particles at a loading of 1 wt%, yielding an average compressive modulus of 3349 ± 36 MPa and a yield stress of 144 ± 0.94 MPa, with a Poisson’s ratio of 0.30 ± 0.046. While significant enhancements were observed, these values are still slightly inferior to the compression modulus of the most highly toughened commercial epoxy (EP2400, D) in the current work, although the yield strength was consistently higher with the presence of the silica nanoparticles. In a similar study, Soutis et al. [9] studied the effect of silica nanoparticles on compressive properties of a commercial epoxy based on the diglycidyl ether of bisphenol A (Epikote 828) when cured with 1-methyl-5-norbornene-2,3-dicarboxylic anhydride. For a comparable cylindrical specimen (tested according to ASTM D695), the following parameters were obtained: compressive modulus 3020 ± 60 MPa, yield stress 133 ± 0.2 MPa, yield strain 6.50 ± 0.05. Once again, these parameters are in line with the specimens tested in the current work.

The various formulations tested in this study involve the screening of different toughening agents in the four commercial epoxy systems to exploit a variety of toughening mechanisms [26]. Although the exact nature of the toughening agents used in the materials in the present study are proprietary, common candidates for this purpose include terminally-functionalised (telechelic) engineering thermoplastics which are capable of forming covalent bonds with the matrix [27] as well as participating in a range of non-covalent interactions (e.g., π-π stacking where both the matrix and the toughening agent contain aromatic moieties). Other notable mechanisms to facilitate strong interfaces between the matrix and toughening agent include electrostatic interactions, where strongly polar bridging species are present, or through the formation of hydrogen bonds [28], and dispersion forces such as van der Waals’ associations, which become more influential in aliphatic polymer matrices [29]. For example, the use of an epoxy-compatible thermoplastic toughening agent in the epoxy matrix CYCOM^®^977-2 (B) leads to a reduction in the compression modulus and an increase in compression stress values across the entire stress–strain plot, including the compression yield stress value, compared to the baseline matrix CYCOM^®^890 (A). In contrast, the dispersed toughening particles in PR520 (C) increase the compression modulus, with virtually no change in the measured compressive yield stress. However, past the yield point, compression stress values remained consistently lower than those observed for the baseline epoxy matrix CYCOM^®^890 (A); this observation is consistent with the network architecture and lower crosslink density developed by PR520 (C) [30,31]. 

Finally, the toughening agents in the PRISM EP2400 epoxy matrix (D) lifted all compression stress values, past the yield point, back to the values previously observed with the CYCOM^®^890 matrix (A). Despite a slight decrease in the compression modulus, the addition of a thermoplastic toughening agent provided the most noticeable increase in compressive stress values at and past the yield point of the matrix. This toughening strategy appears as highly desirable in applications where higher compression performance might be required.

### 3.3. Dilation Angle

**Figure 7:**
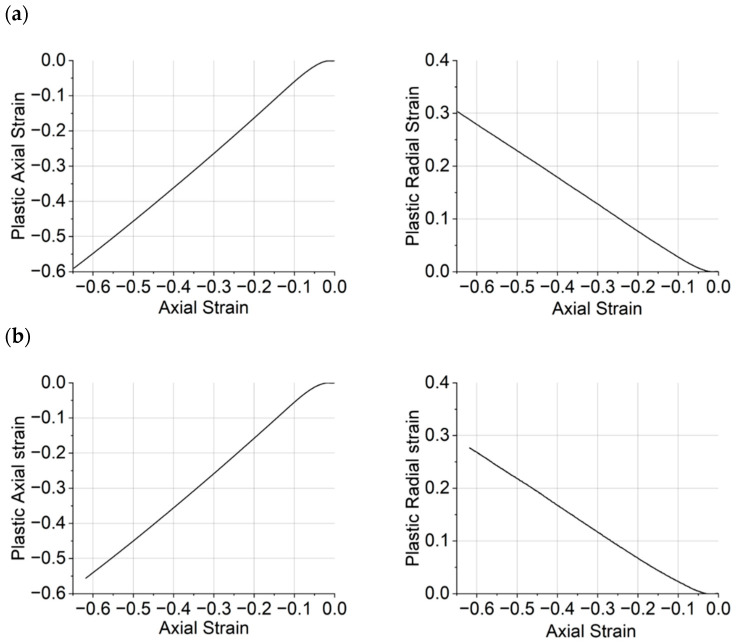

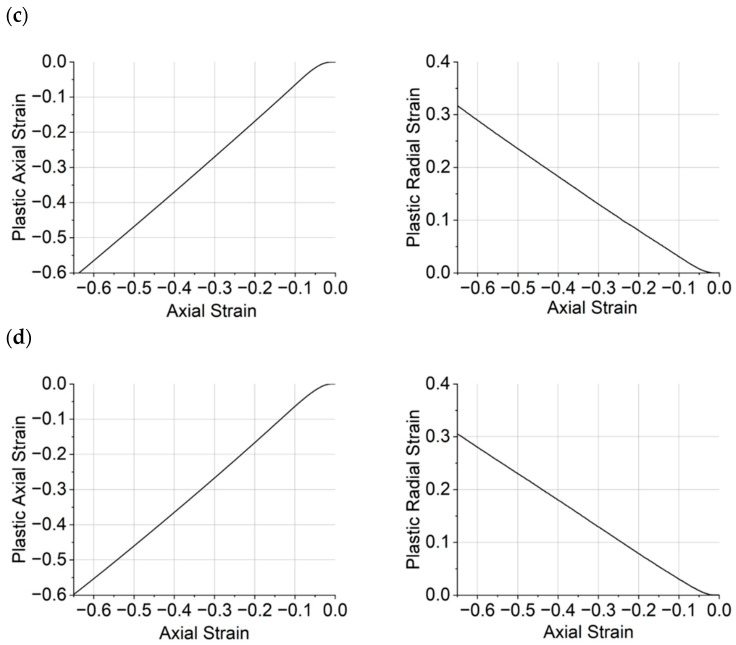
Plastic axial strain vs. axial strain (left) and plastic radial strain vs. axial strain (right) for (**a**) CYCOM^®^890, (**b**) CYCOM^®^ 977-2, (**c**) PR520, and (**d**) PRISM EP2400.

A similar methodology was adopted to determine the dilation angle to that reported in [32], and the dilation angle was calculated from Equation (2). Representative radial and axial components of plastic strain vs. total axial strain obtained from the epoxy resin samples are shown in Figure 7. Initially, both plastic axial and radial components are zero during the linear regime (depicted as I in Figure 5), where there is no plasticity. After an initial non-linear region (Figure 5, II), both components follow an almost linear trend with respect to the total axial strain, approximately starting from the beginning of the softening regime (Figure 5, III). The strain hardening stage (Figure 5, IV) is marked by the similarity in response observed for CYCOM^®^890 (A), CYCOM^®^977-2 (B), and PRISM EP2400, which share similar base resin structures, although the greatest stress is recorded for CYCOM^®^977-2 (B). In contrast, PR520 (C) displays a distinctly different yielding behaviour and little strain hardening, consistent with a more open, less crosslinked network [30,31]. An annotated diagram is presented in Figure S3 for CYCOM^®^890 (A), showing the softening region.

**Figure 8:**
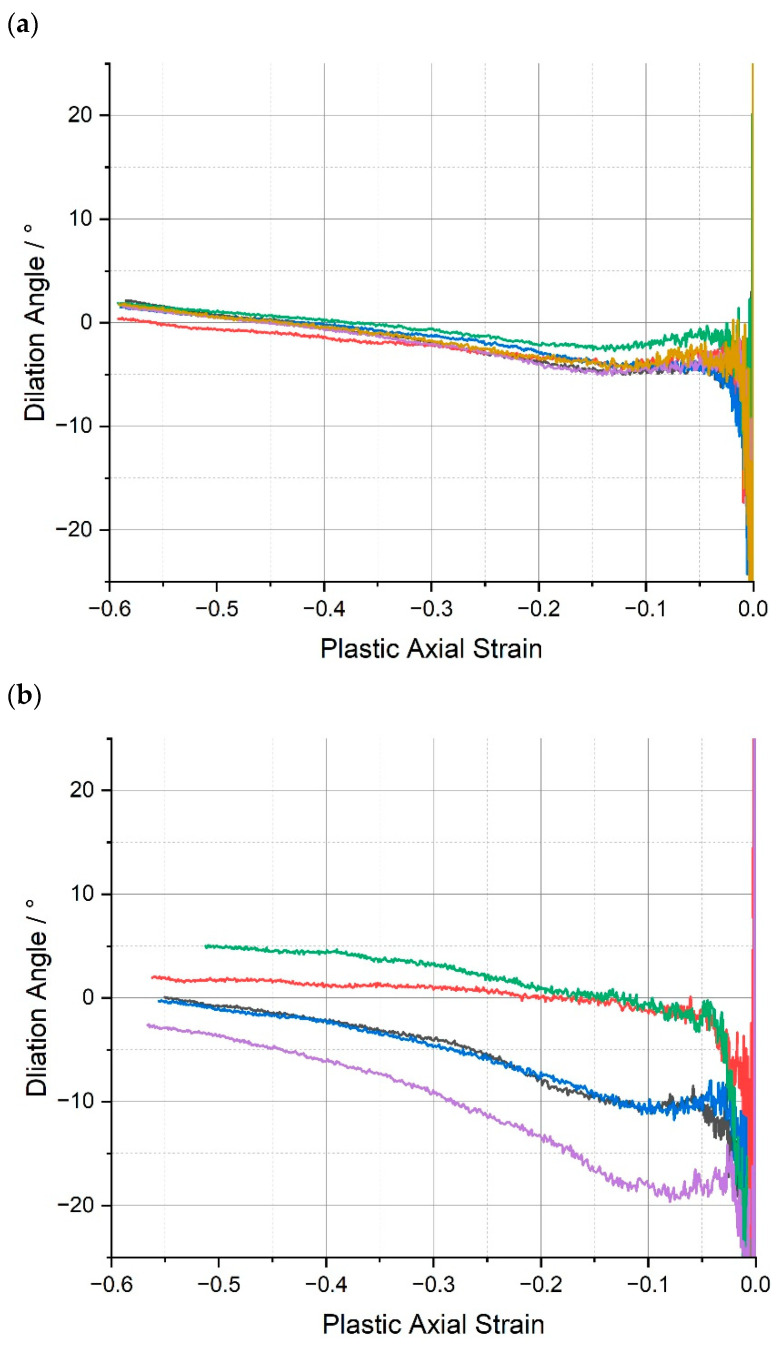

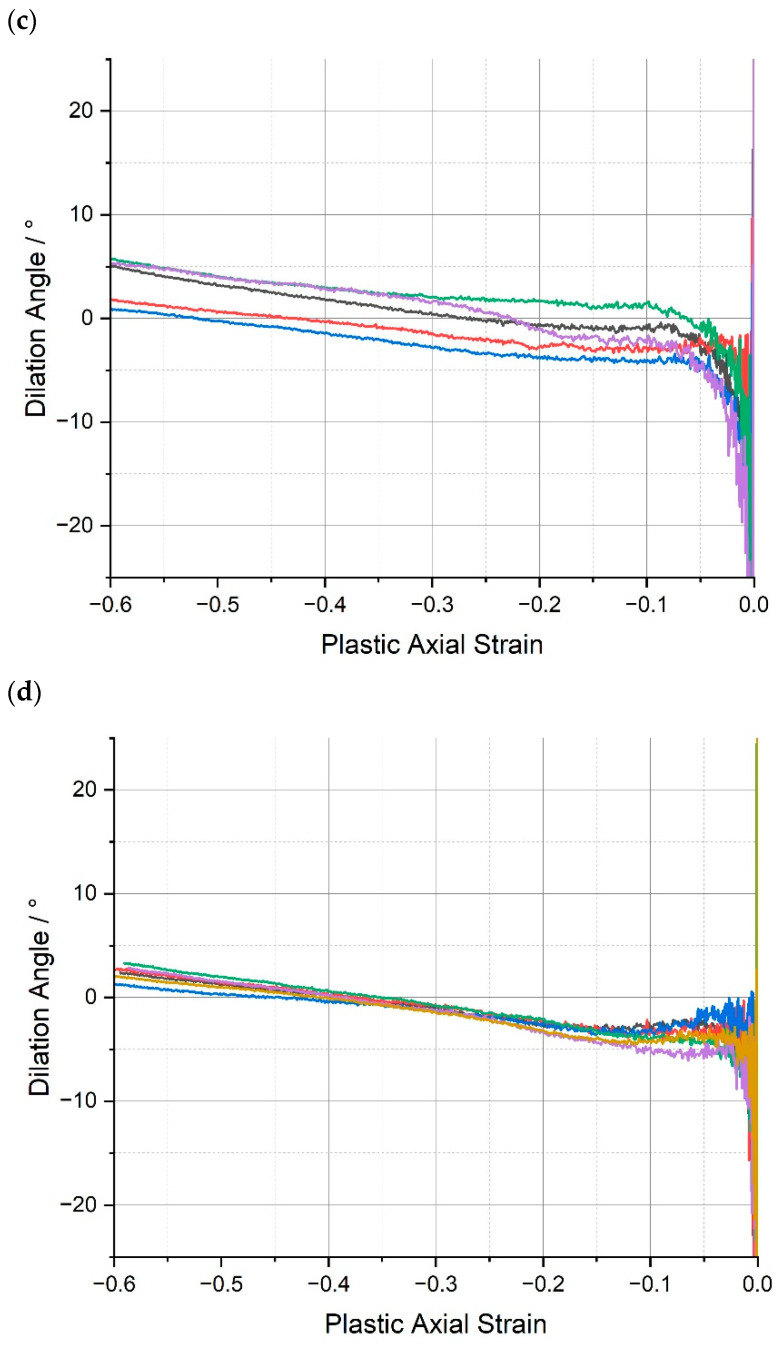
Dilation angle, ψ, vs. plastic axial strain, $e_{p}^{z}$, for (**a**) CYCOM^®^890, (**b**) CYCOM^®^ 977-2, (**c**) PR520, and (**d**) PRISM EP2400. Replicate analyses of same material shown by different colours.

In this work, the dilation angle is presented as a function of the plastic axial strain, recognising that the dilation angle is only truly applicable when plasticity is present. The dilation angles vs. plastic axial strain, $e_{p}^{z}$, for the range of epoxy resins, are shown in Figure 8.

In general, there is significant amount of scatter in the values of the dilation angle for the epoxies tested at low plastic strains and stabilisation after a plastic strain of approximately 0.15–0.2, after which is seems to increase linearly with respect to the axial strain (although this is harder to discern in the case of CYCOM^®^ 977-2 (B)). Consequently, in common with our previous publication, for the purposes of comparison, it was decided to select a representative value of the dilation angle at a plastic strain of 0.3 to ensure that full softening had occurred, because the dilation angle does not appear to yield a stable value at small plastic strains or even within part of the softening region (Figure S4). Thus, the average values for the plastic Poisson’s ratio and the dilation angles of the epoxy resins are presented in Table 3.

The average dilation angles of the epoxy resins measured at the plastic strain at 0.3 all fall close to 0°. The dilation angle values presented in Table 3 suggest that at such strain, plastic deformation occurs almost at a constant volume, similar to what was observed in ref. [32] for an untoughened resin. In addition, from Figure 8, considering the data scatter in dilation angle vs. plastic axial strain curves, no significant statistical difference can be identified across the different material systems. The dilation angle data available for direct comparison are relatively limited; however, perhaps the HexFlow^®^ RTM6 (similarly a one-component, untoughened epoxy resin designed for infusion processing) provides the most suitable candidate and is the subject of several studies. Morelle et al. [10] measured the average dilation angle of RTM6 epoxy resin (in compression) and reported it to be 0°. Sorini et al. [33] calibrated their constitutive model accounting for tension/compression asymmetry in RTM6 and reported different values of dilation angle under tension and compression: 14.28° and −0.001°, respectively.

**Figure 9:**
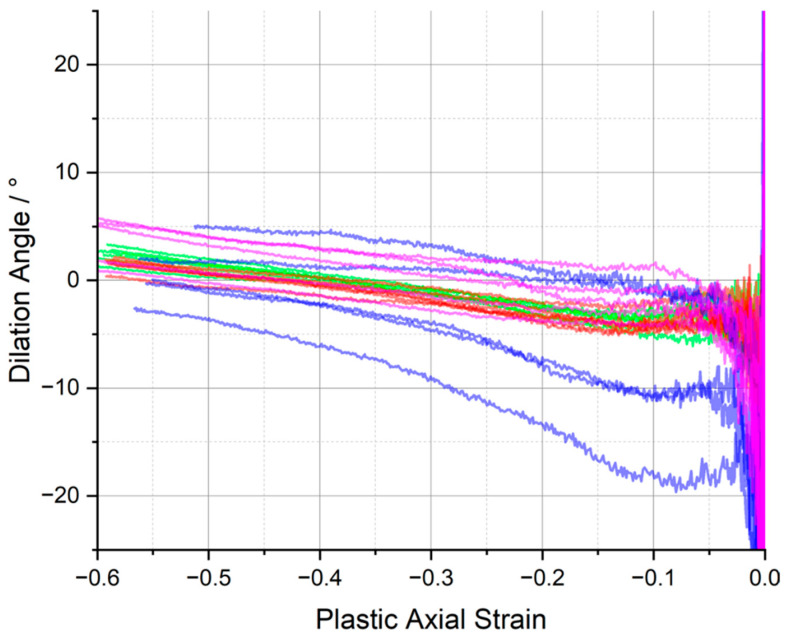
Dilation angle, ψ vs. plastic axial strain for CYCOM^®^890 (red), CYCOM^®^ 977-2 (blue), PR520 (pink), and PRISM EP2400 (green).

Across the whole range of the tested toughened epoxy matrices, CYCOM^®^977-2 (B), PR520 (C), and PRISM EP2400 (D), or the baseline CYCOM^®^890 (A), Poisson’s ratio remained virtually unchanged, typically at 0.46, and the ultimate dilation angle remained near 0°, although the greatest spread in the data, −19° to +5°, was observed for CYCOM^®^977-2 (B), presumably reflecting the effect of the thermoplastic toughening agent (Figure 9). The consistency in these two values across all four tested resin systems demonstrates that the intrinsic physical properties of the crosslinked epoxy matrices were very similar despite the differences in the composition of the resin systems and were independent of the type of toughening agents that were added to the various tested epoxy formulations. This absence of significant changes in dilation angle and Poisson’s ratio is most likely due to the use of relatively modest amounts of toughening agents being incorporated in these epoxy formulations, which was confirmed by very similar thermogravimetric data being recorded across all four of the tested matrices. Furthermore, this relatively low value for dilation angle across all four of the tested materials showed that these materials underwent relatively negligible volume increase or ‘dilatancy’ during the compression-induced plastic flow or deformation.

**Figure 10:**
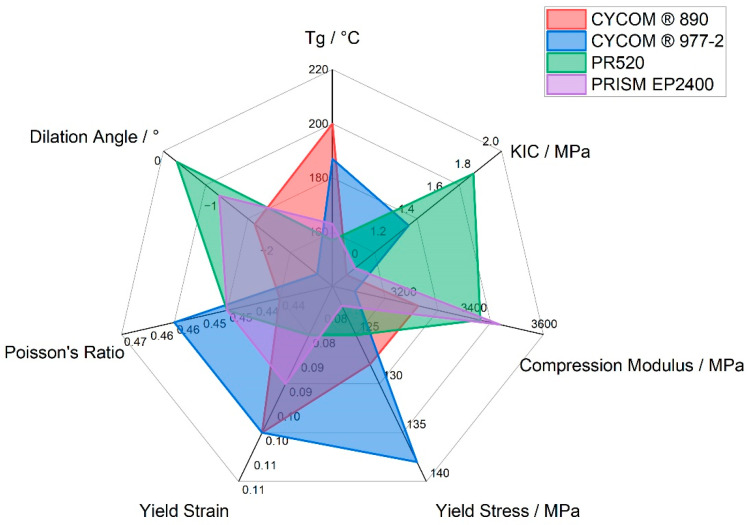
Radar plot showing selected physical and mechanical data for CYCOM^®^890, CYCOM^®^ 977-2, PR520, and PRISM EP2400.

The variation in plastic dilatancy observed between the different epoxy resins may be related to differences in molecular architecture and internal forces. The highly crosslinked networks produced in CYCOM^®^890 (A), CYCOM^®^977-2 (B), and PRISM EP2400 (D) will all produce significant concentrations of hydrogen bonds to restrict molecular mobility and limit segmental motion. Molecular dynamics simulations show that polymer networks bound by stronger interchain associations will exhibit smaller volume dilation [15]. The more open the network produced in cured PR520 (C), with a lower crosslink density, offers more molecular freedom to undergo molecular rotation; hence, the apparent lower dilation angle but higher *K_IC_* value. These data, along with the combined physical and mechanical data for all four cured epoxy resin systems, are shown in Figure 10.

## 4. Conclusions

Commercially available, amine-cured, epoxy matrices were characterised by DSC, TGA, and SEM, and mechanically tested in quasi-static uniaxial compression. The addition of a functionalised non-phase-separating thermoplastic toughening agent into the cured, highly crosslinked epoxy resin network preserved the glass transition temperature of the untoughened epoxy matrix, while increasing the measured true axial stress values across the whole stress–strain curve, at the cost of a relatively minor drop in the compression modulus of 6% compared to a baseline amine-cured epoxy matrix. The combination of toughening agents in an epoxy matrix, PRISM EP2400 (D,) brought the overall compression performance in the plastic region of the material back to that observed for the baseline material, while maintaining the 5% increase in the compression modulus, obtained with the sole addition of toughening particles into the epoxy resin matrix. The mechanical properties and the overall stress–strain response in compression may be used to parameterise computational models of real composites in compression. However, the performance of these fully formulated engineering resins is in the same range as the ‘model’ systems reported previously. The compression stress–strain curves in the literature are, therefore, confirmed to be representative of the performance of real systems. All four epoxy resins demonstrated relatively low values for dilation angle but volume increases, or ‘dilatancy’, during the compression-induced plastic flow or deformation were primarily influenced by the polymer architecture (i.e., crosslink density) and inter-molecular associations offered by the toughening agents. 

## Figures and Tables

**Table 1:** STA (DSC + TGA) data summary for cured epoxy matrices.

Name	*T_g_*, °C	*T_dec_*, °C	Mass Loss, %
CYCOM^®^890 (A)	200	373	82
CYCOM^®^977-2 (B)	187	377	72
PR520 (C)	157	372	79
PRISM EP2400 (D)	163	377	85

Key: *T_g_* = glass transition temperature determined from mid-point of discontinuity in DSC response, decomposition temperature (*T_dec_*) determined at the intersection of tangents at the onset of mass loss, with mass loss calculated as the total mass % change between 50 and 800 °C.

**Table 2:** Average and standard deviation values for the compression properties of the tested epoxy matrices.

	Compression Modulus	Yield Stress	Yield Strain
Name	*E*, MPa	*s_y_*, MPa	*e* *_y_*
CYCOM^®^890	3244 ± 74	128 ± 1	0.102 ± 0.002
CYCOM^®^977-2	3064 ± 36	138 ± 1	0.101 ± 0.006
PR520	3421 ± 99	125 ± 1	0.076 ± 0.003
PRISM EP2400	3476 ± 97	122 ± 1	0.092 ± 0.002

**Table 3:** Average values for the Poisson’s ratio and the dilation angles of the tested epoxy matrices.

	Poisson’s Ratio	Dilation Angle
Name	*ν*	$\psi (\varepsilon_{p}\;=\;0.3)$, °
CYCOM^®^890	0.44 ± 0.01	−1.62 ± 0.59
CYCOM^®^977-2	0.46 ± 0.01	−2.73 ± 4.8
PR520	0.45 ± 0.01	−0.24 ± 2.05
PRISM EP2400	0.45 ± 0.01	−0.99 ± 0.26

## Data Availability

Data will be made available on request.

## Supplementary Materials

The following are available online at https://www.mdpi.com/article/10.3390/polym15194022/s1, Figure S1: Differential scanning calorimetry data plotted as heat flow (exothermic flow upwards) versus temperature for the baseline epoxy resin CYCOM^®^890 (A), Figure S2: Thermogravimetric analysis data plotted as residual mass (%) versus temperature for the cured baseline epoxy resin CYCOM^®^890 (A), Figure S3: Plastic axial strain vs. axial strain (top) and plastic radial strain vs. axial strain (bottom) for CYCOM^®^890, showing softening region, Figure S4: Dilation angle vs. plastic axial strain for CYCOM^®^890, showing softening region.
