# Supplementary material for: Examining the Quasi-Static Uniaxial Compressive Behaviour of Commercial High-Performance Epoxy Matrices
Source: Polymers (Basel). 2023 Oct 8;15(19):4022. doi: 10.3390/polym15194022 (PMC10574947; doi:10.3390/polym15194022)
Supplement: Supplementary file 1 [file polymers-15-04022-s001.zip › polymers-2546403-supplementary.pdf]

Examining the Quasi-Static Uniaxial Compressive Behaviour of Commercial High-Performance Epoxy Matrices

J. F. Gargiuli [1], G. Quino [1,2], R. Board [1], J. C. Griffith [1], M. S. P. Shaffer [3], R. S. Trask [1] and I. Hamerton [1,*]

Supplementary information

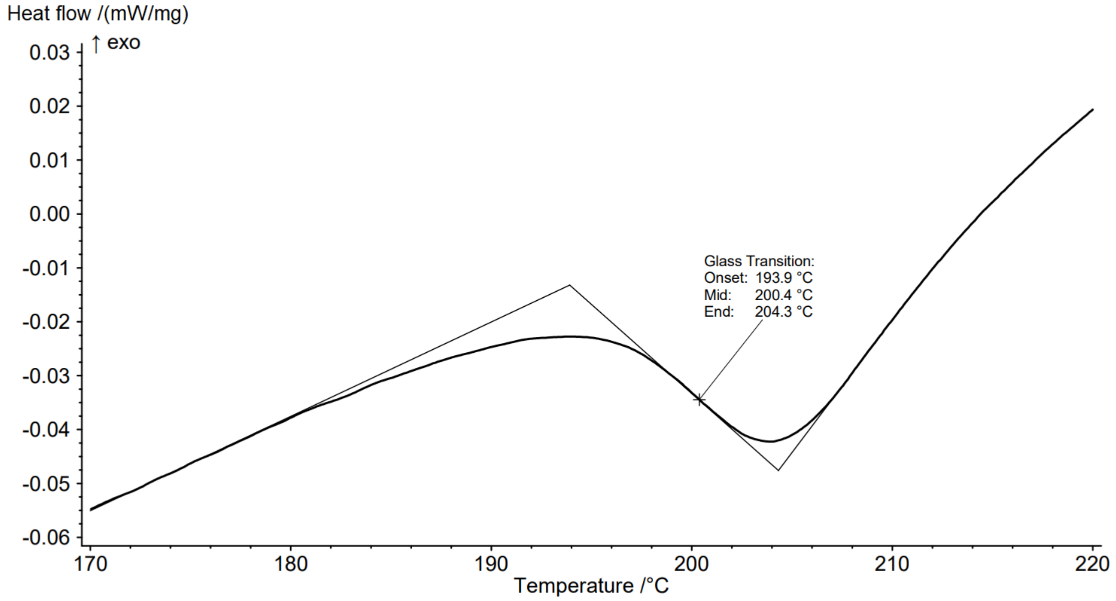

Figure S1. Differential scanning calorimetry data plotted as heat flow (exothermic flow upwards) versus temperature for the baseline epoxy resin CYCOM®890 (A)

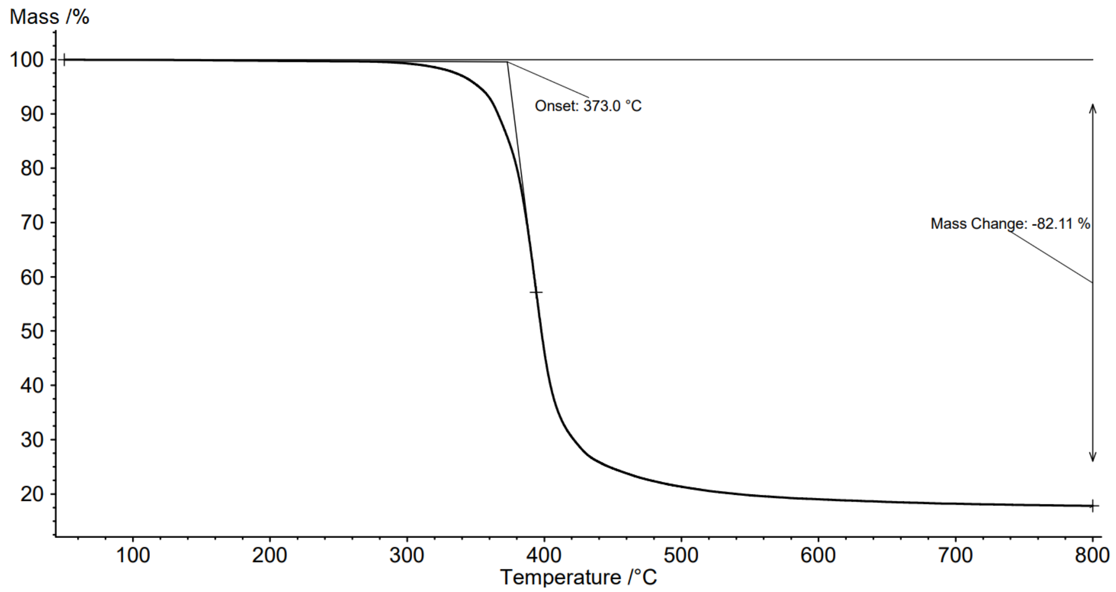

Figure S2. Thermogravimetric analysis data plotted as residual mass (%) versus temperature for the cured baseline epoxy resin CYCOM®890 (A)

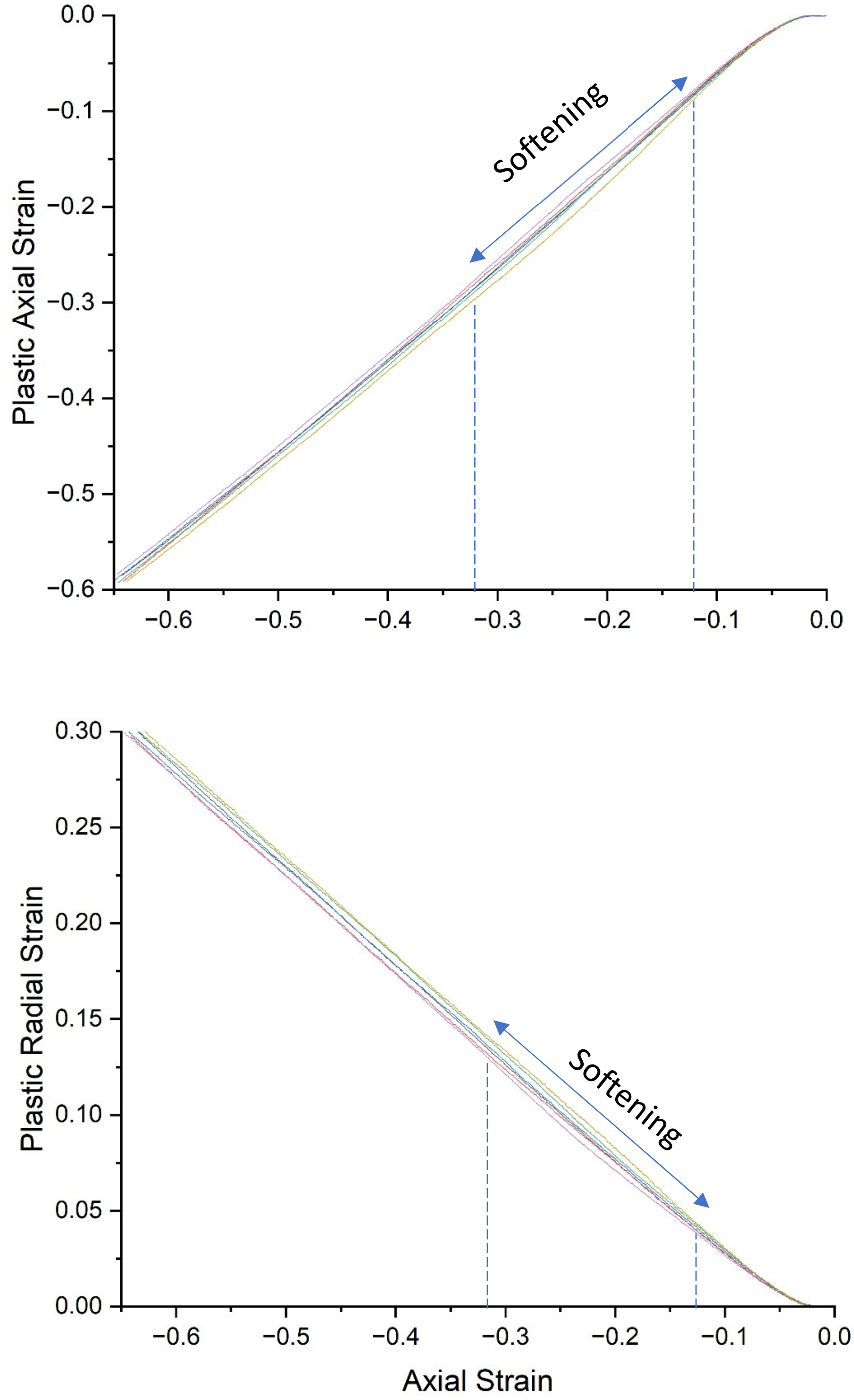

Figure S3. Plastic axial strain vs. axial strain (top) and plastic radial strain vs. axial strain (bottom) for CYCOM®890, showing softening region.

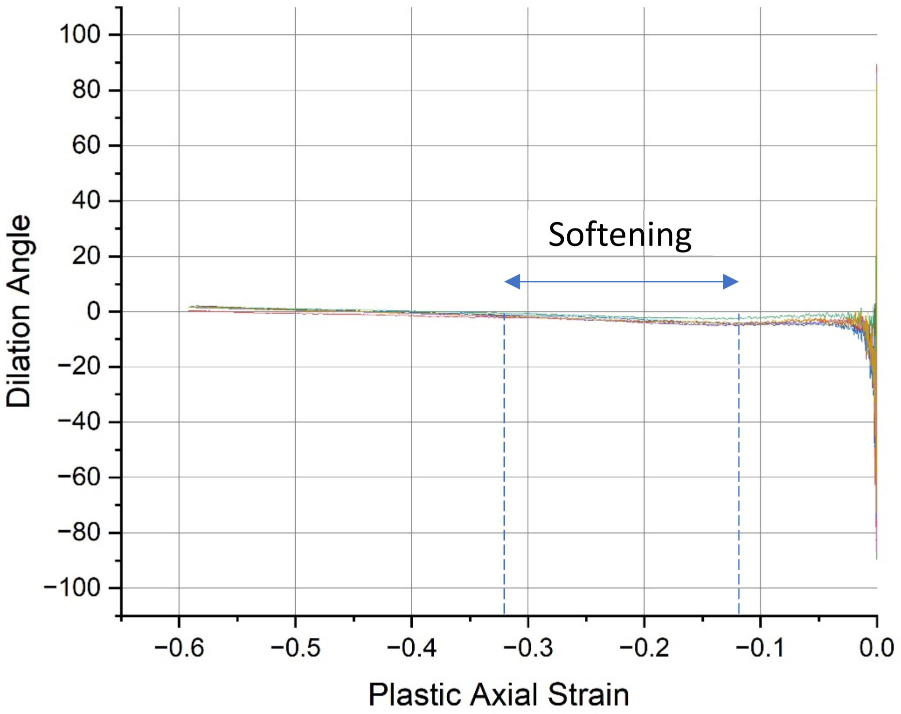

Figure S4. Dilation angle vs. plastic axial strain for CYCOM®890, showing softening region.
